# An integrative approach for imaging and quantitative analysis of gut microbiota growth *in vivo* using fluorescent D-amino acid labeling and fluorescence *in situ* hybridization

**DOI:** 10.52601/bpr.2024.240044

**Published:** 2025-06-30

**Authors:** Yingjun Zhou, Liyuan Lin, Wei Wang

**Affiliations:** 1 State Key Laboratory of Genetic Engineering, Department of Microbiology, Fudan Microbiome Center, School of Life Sciences, Fudan University, Shanghai 200433, China; 2 Institute of Molecular Medicine (IMM), Renji Hospital, Shanghai Jiao Tong University School of Medicine, Shanghai 200082, China

**Keywords:** Gut microbiota, Fluorescent D-amino acid, Fluorescence *in situ* hybridization, Bacterial growth and division, Metabolic status

## Abstract

The profound influence of gut microbiota on human health has been well-recognized; however, substantial gaps remain in our understanding of the highly diverse and dynamic processes of microbial growth and activities in the gut. Conventional methods, which primarily rely on DNA sequencing, provide limited insights into these aspects. This paper presents a protocol that integrates fluorescent D-amino acid (FDAA) metabolic labeling with fluorescence *in situ* hybridization (FISH) for imaging and quantitatively analyzing the *in vivo* growth of gut microbiota. By administering two FDAAs sequentially through mouse gavage, we label the peptidoglycan of gut bacteria in their native environment, allowing the labeling signals on bacterial cell walls to serve as markers of cellular proliferation and division. We have also demonstrated that the intensity of FDAA labeling directly correlates with the metabolic activity of gut bacteria. Additionally, FISH is employed to distinguish specific bacterial taxa of interest via fluorescence microscopy or flow cytometry. This integrative method greatly enhances our capacity to visualize and measure the *in vivo* growth and metabolic states of various gut bacteria, thereby illuminating the previously obscured “dark matter” in the gut ecosystem.

## INTRODUCTION

In recent years, the relationship between gut microbiota and human health has attracted enormous attention from different research fields (Schmidt *et al.* 2018). The growth, division, and metabolic activities of microbes within the gut can influence their functional impacts on the host (Almeida *et al.* 2019). While DNA sequencing technologies have dramatically improved our comprehension of the gut microbiota, there are still limitations when attempting to decipher microbial functions solely on genetic data (Fricker *et al.* 2019). Culturomics has advanced the field by facilitating the *in vitro* cultivation of a broader spectrum of gut bacteria. However, it is important to realize that the cultivation of many microbial species remains a huge challenge, and findings from *in vitro* studies may not fully recapitulate the complexities of the *in vivo* environments (Rinke *et al.* 2013; Schmidt *et al.* 2018). Consequently, there is an urgent need in gut microbiota research for new methodologies that can directly investigate the *in vivo* behaviors of gut bacteria, including their growth, division, and metabolism, to achieve a comprehensive understanding of their roles in both health and disease.

To study a complex biological system like the gut microbiota, the metabolic labeling technique using unnatural probes has some unique advantages because of their ease of use, avoidance of genetic engineering, and the ability to directly reflect certain metabolic processes and activities of bacteria. Fluorescent D-amino acid (FDAA) probe, which can label bacterial peptidoglycan (PGN) with high specificity, coverage, and efficiency, is an ideal tool for gut microbiota research (Hudak *et al.* 2017; Wang *et al.* 2019). The incorporation of FDAA probes into bacteria cell wall is mediated by the activities of PGN transpeptidases, including both D, D- and L, D-transpeptidases, which are responsible for forming cross-links between stem peptides in PGN synthesis (Kuru *et al.* 2019). During cross-linking, D, D- and L, D-transpeptidases first attack the amide bonds between the 4^th^ and 5^th^ amino acid or 3^rd^ and 4^th^ amino acids of the stem peptide, and form enzyme-substrate intermediates via an ester or thioester bond, respectively. These intermediates can be attacked by the amine in FDAAs and then form new amide bonds between FDAA and the stem peptide, thus replacing the original 4^th^ or 5^th^ amino acid with the fluorescent probe (Fura *et al.* 2015; Hsu *et al.* 2019; Kuru *et al.* 2012). Moreover, we found that the activity of these transpeptidases could reflect the basic growth status of bacteria (Lin *et al.* 2020a); therefore, the faster the growth and metabolic rate of a specific bacterium, the more active its cell wall synthesis, resulting in a stronger FDAA fluorescence labeling signal (Lin *et al.* 2021a; Lin *et al.* 2020a). This offers a means to assess the native metabolic levels of gut bacteria. To obtain taxonomic information of the labeled bacteria, fluorescence *in situ* hybridization (FISH) is used, which utilizes fluorescent DNA probes with a high degree of sequence complementarity to specifically bind characteristic 16S rRNA within a bacterial cell (Delong *et al.* 1989).

By combining these techniques, we can directly obtain information about the native status of specific bacterial groups, as identified by FISH, through the *in vivo* labeling provided by FDAAs. For instance, this approach enables us to observe how the metabolic levels of a particular bacterial species are influenced by various endogenous or exogenous factors. By sequentially introducing two distinct FDAA probes via gavage, we can discern the *in situ* growth and division patterns of various gut bacteria. This is because the first probe has a lower concentration than the second owing to the host absorption. The difference results in labeling cell walls of different bacteria with distinct patterns, where more actively constructed PGN sites (*e*.*g*. septum) would have stronger labeling signals of the second probe. Here, we detail the technical aspects of this strategy for assessing the growth and metabolic status of gut microbiota. This approach is expected to greatly expand our understanding of the intricate *in vivo* dynamics of the gut microbiome.

## MATERIALS

### Reagents and materials

• FAM-amino-D-alanine (FADA, Chinese Peptide Company)

• TAMRA-amino-D-alanine (TADA, Chinese Peptide Company)

• Cy5-amino-D-alanine (Cy5ADA, Chinese Peptide Company)

• Formamide (Sigma-Aldrich, Cat. F9037)

• Phosphate buffered saline (Thermo Fisher Scientific, Cat. 10010023)

• 4% paraformaldehyde (Sangon Biotech, Cat. E672002)

• Acetonitrile (Sigma-Aldrich, Cat. 360457)

• TE buffer (Sangon Biotech, Cat. B548106)

• NaCl (Sangon Biotech, Cat. A610476)

• Tris-HCI buffer (Thermo Fisher Scientific, Cat. 15567027)

• 10% SDS Solution (Sangon Biotech, Cat. B548118)

• 20× Saline sodium citrate (SSC) buffer (Solarbio, Cat. S1030)

• 50× Denhardt's solution (Solarbio, Cat. D1080)

• Salmon sperm DNA (Solarbio, Cat. H1060)

• Lysozyme (Solarbio, Cat. L1080)

• Agarose (Sangon Biotech, Cat. A600234-0025)

• EtOH (Sangon Biotech, Cat. A500737)

• Cell Strainer, 40 μm (Sangon Biotech, Cat. F613461)

• FISH probe (Sangon Biotech)

### Animals

• 6−8 weeks C57BL/6 male mice, SPF (GemPharmatech)

### Equipment and software

• ThermoMixer (Eppendorf)

• CytoFLex (Beckman Coulter Life Sciences)

• TCS SP8 laser scanning confocal microscope (Leica)

• Huygens Essential Deconvolution software (Scientific Volume Imaging B.V., Hilversum)

## PROCEDURE

### *In vivo* labeling of gut microbiota with FDAA probes

1 The FDAA probe powder was prepared in a 10 mmol/L stock solution of 20% acetonitrile in H_2_O, and stored at –30°C for long-term preservation. Repeated freeze-thaw cycles should be avoided.

2 The C57BL/6 mice were gently intragastrically administered with 200 μL of FDAA (1 mmol/L in distilled H_2_O).

**Figure 1:**
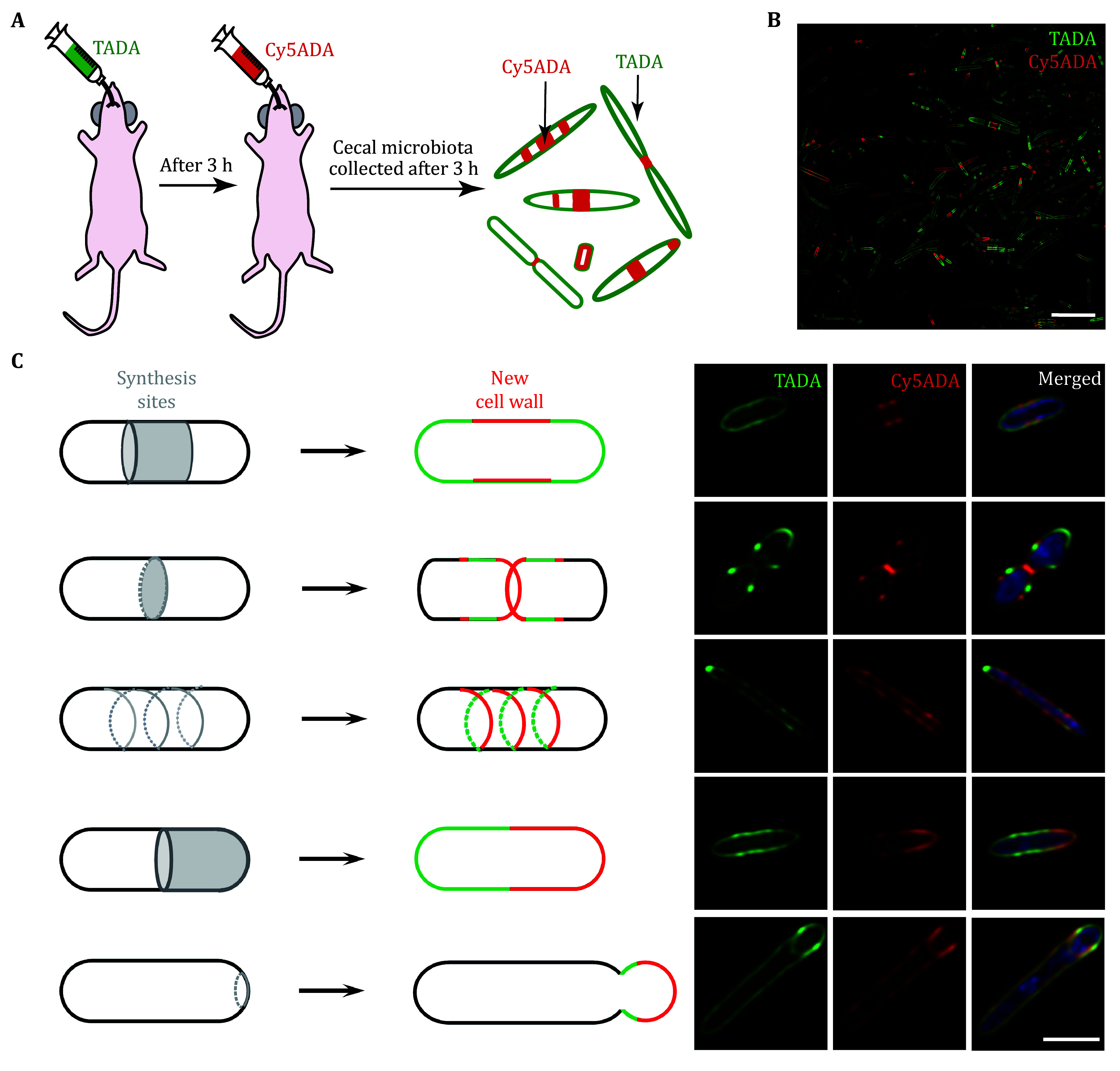
The classical bacterial growth and division patterns observed in the labeled gut microbiota. **A** Schematic illustration of the workflow. C57BL/6 mice were intragastrically administered with TADA and Cy5ADA with a 3 h interval, and cecal microbiota was harvested in 3 h following the second gavage. **B** Cecal microbiota labeled by STAMP analyzed by confocal microscopy. Scale bar, 10 μm. **C** Illustration of the classical bacterial growth and division patterns. In the cartoon, gray regions represent the sites of active cell wall synthesis; green and red indicate the newly constructed PGN during the STAMP labeling process. The right panel shows the bacteria with the corresponding division patterns observed in the cecal microbiota. Scale bar, 2 μm

3 If sequential labeling is required, there should be a 3 h interval between the gavage of the two FDAA probes. We refer to this strategy as STAMP (Sequential Tagging with D-Amino acid-based Metabolic Probes); schematic illustration and imaging data of the STAMP-labeled cecal microbiota are shown in Figs. 1A and 1B. It can reflect the growth and division of microbiota in the gut through the distribution of two signals on the cell wall. Five previously known bacterial division patterns could all be found in the labeled gut microbiota (Fig. 1C).

**[Tips]** Of note, for bacteria having very fast or slow growth rates, the 3 h time interval can be adjusted accordingly.

4 Three hours after the last gavage, the gut microbiota labeled with FADA were collected:

(A) The mice were sacrificed by cervical dislocation after anesthesia, and the cecum was dissected and placed in 2 mL of PBS.

(B) Then the mouse cecum was finely minced with a pair of 4.5-inch iris scissors.

(C) To better collect the gut bacteria, it is also necessary to repeatedly pipette the liquid with a 1 mL wide-orifice pipette tip.

(D) The minced tissues and digesta were filtered through a 40 μm cell strainer (1500*g*, 2 min) to separate most of the non-bacterial tissue debris from the bacteria.

(E) The bacterial pellets were then washed twice with 1.5 mL PBS by centrifugation (15,000*g*, 3 min), and resuspended in PBS to reach an appropriate concentration for subsequent experiments.

### Fluorescence *in situ* hybridization (Fig. 2A)

**Figure 2:**
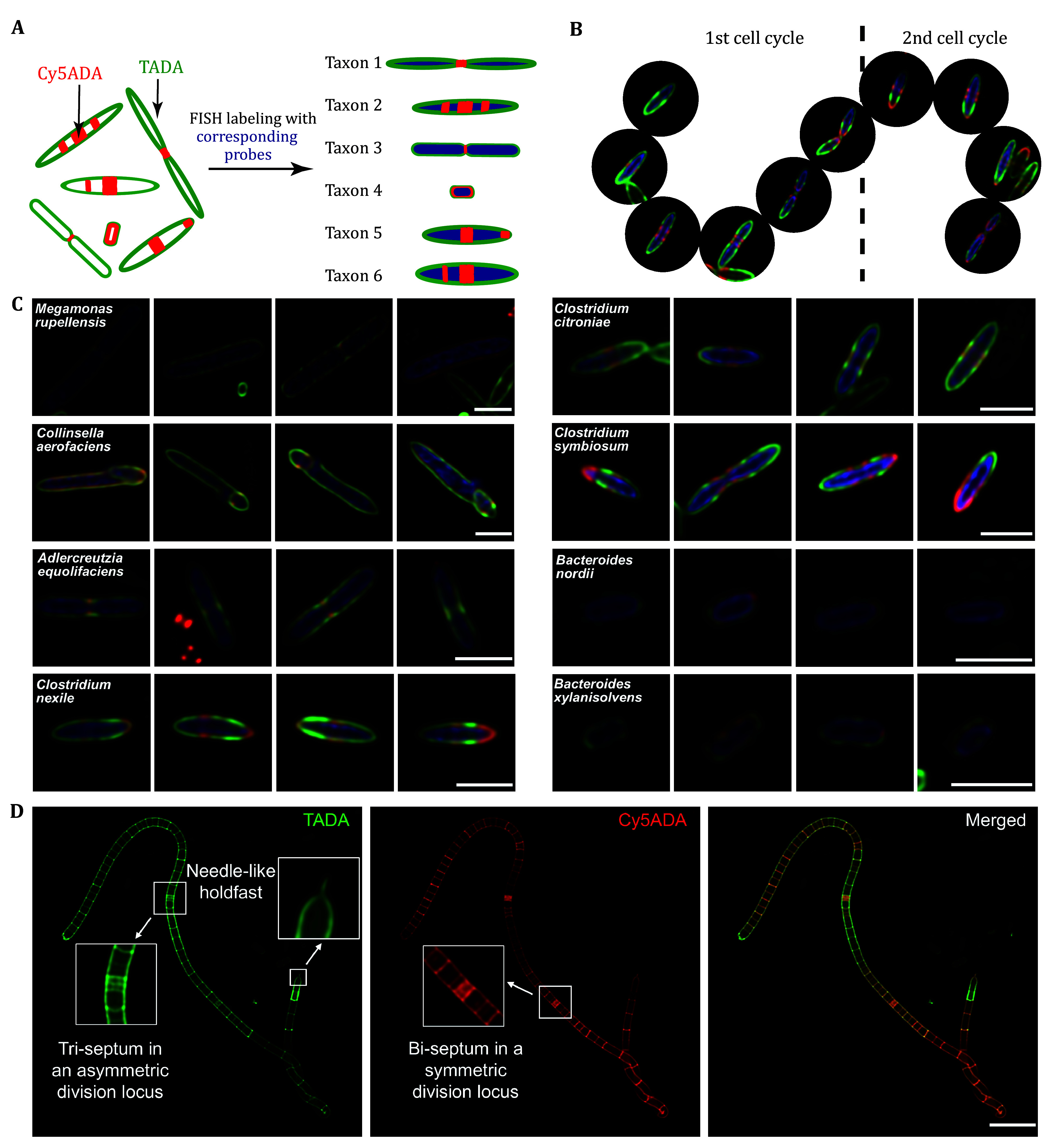
The integrative use of STAMP and FISH to visualize the *in vivo* growth and division patterns of bacteria in the gut microbiota. **A** Schematic illustration of the workflow. **B** STAMP recorded the PGN construction and remodeling of *C. symbiosum* cells growing at different stages during two cell cycles. **C** Bacterial cell morphologies and STAMP-labeling patterns in each FISH-tagged bacterium. Scale bar, 2 μm. **D** Confocal fluorescence imaging of the STAMP-labeled SFB. Scale bar, 5 μm

5 Routine metagenomic sequencing was performed on bacterial samples to determine their compositions for subsequent FISH staining.

6 Before proceeding with FISH labeling, the fixation and permeabilization of microbiota are crucial:

(A) An equal volume of 4% paraformaldehyde fix solution was added to the microbiota suspension and the sample was then incubated at room temperature for 1.5 h to fix the microbiota. Optimal incubation results are achieved by using a shaking platform.

**[CAUTION!]** Paraformaldehyde is toxic and carcinogenic. All sample preparations involving paraformaldehyde should be performed in a chemical hood.

(B) After the fixation, the sample was washed twice with PBS and resuspended in EtOH-PBS (1:1, *v*/*v*) buffer, which was then stored at −20°C for at least 24 h before the following experiment.

7 Subsequently, an appropriate amount of the fixed microbiota solution was taken for the FISH experiment, followed by centrifugation to remove the stocking buffer residue.

8 The microbiota was resuspended in a hybridization buffer (0.9 mol/L NaCl, 20 mmol/L Tris (pH 7.5), 0.01% SDS), and formamide if required (Table 1).

**Table 1:** FISH probes used in this work and their staining conditions (Lin *et al.* 2021b)

Target bacteria	Probe	Probe sequence （5’-3’）	Hybridization Temp. (°C)	Formamide Conc. (%, *v*/*v*)
*Clostridium nexile*	Cnexi59	TGT TAC GAC TTC ACC CCA GTT ATC GGT C	46	45
*Collinsella aerofaciens*	Caero1378	TCT CGG TTG GGC CGG CGA CTT CGG GTG C	46	20
*Adlercreutzia equolifaciens*	Aequo1433	GTT ACG ACT TCA CCC CCC TTA CCC TCC A	46	55
*Bacteroides nordii*	Bnord148	ATC CCA TGC GGA AAT ATT ATA CCA TCG G	46	20
*Bacteroides xylanisolvens*	Bxyla686	ATC AGT GTC AGT TGC AGT CTA GT	46	55
*Megamonas rupellensis*	Mrupe35	CGC GTT ACT CAC CCG TTC GCG CAC T	46	55
*Clostridium citroniae*	Ccitr947	AGG TCA CTT TAC TGA CCG GTC AGG G	46	20
*Clostridium symbiosum*	Csymb627	CCG ACA CTC CAG TTA AAC AGT TTC C	46	20

9 The DNA probe for FISH was added to the sample with a final concentration of 5 ng/µL and incubated overnight at the required temperature (Table 1) in a ThermoMixer.

10 After hybridization, the microbiota was then washed (2 × 15 min) at the required washing temperature in washing buffer (0.9 mol/L NaCl, 20 mmol/L Tris (pH 7.5), 0.01% SDS).

11 Certain microbes are difficult to permeabilize (such as *Lactobacillus*), and additional processing is required:

(A) After being stored at –20°C for 24 h, the microbiota was resuspended in TE buffer containing 10 mg/mL lysozyme at 37°C for 10 min.

(B) The sample was washed twice with PBS and resuspended in another hybridization buffer (5× SSC, 5× Denhardt, 0.5% SDS, 100 μL/mL salmon sperm DNA) with 5 ng/µL DNA probe and incubated overnight in a ThermoMixer.

**[Tips]** All these reagents (lysozyme, Denhardt, and salmon sperm DNA) must be stored at –20°C and should be prepared and used immediately.

(C) A stringent wash was performed for 2 × 15 min using a buffer containing (1× SSC, 1% SDS).

12 The microbiota was then resuspended in a PBS buffer before analysis with fluorescence microscopy and flow cytometry.

### Confocal fluorescence microscopy

13 The FAM, TAMRA, and Cy5-labeled probes were excited with 488 nm, 552 nm, and 633 nm laser, respectively.

14 A bacterial suspension (1 μL) was added to an agarose pad (1.5% in PBS, ~1 mm thick) (Skinner *et al*. 2013), and then covered with a glass coverslip.

15 Confocal fluorescence imaging was performed on a TCS SP8 laser scanning confocal microscope with a 63x oil immersion lens.

16 Deconvolution of the images was performed using Huygens Essential Deconvolution software using a theoretical point spread function. The identification of specific bacterial species by FISH, combined with the STAMP signals on individual cells, can reveal their *in vivo* growth at different stages during two cell cycles (Fig. 2B). Some of the identified species showing distinct division patterns are shown in Figs. 2C and 2D.

### Flow cytometry analysis

17 The acquisition threshold was set on the forward scatter signals and 20,000−30,000 events were collected for further analysis at the low-flow rate of below 1000 events/s.

18 All the measurement parameters of the microbial cells were acquired as logarithmic signals. The microbiota was displayed with flow cytometry plots of logFSC versus logSSC.

**[Tips]** In the forward and side scatter profiles, gut microbiota forms a cluster that includes bacteria with relatively low values, hence ensuring the accuracy of the gate setting is essential.

19 If FADA is used to label gut microbiota to reflect metabolic activity, FISH probes with Cy5 fluorophore are utilized to label specific bacteria; the different fluorescently labeled bacteria were distinguished on the flow cytometry plots of log FITC versus log APC.

20 The cell population with Cy5 signal represented specific bacteria and reflected their proportion within the gut microbiota. The intensity of the FITC fluorescence signal (median value) from this cell population indicated the metabolic activity.

**Figure 3:**
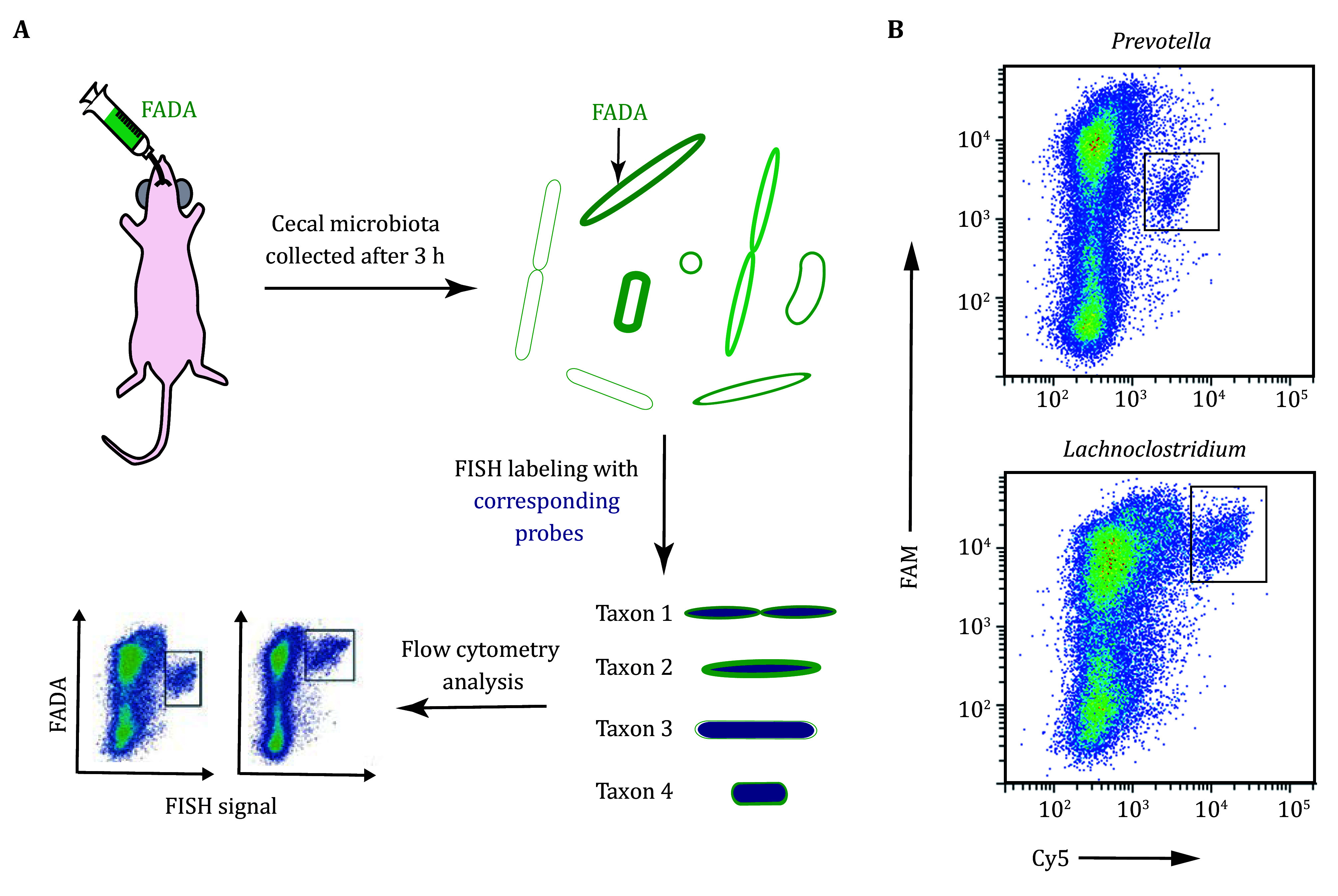
**A** Schematic illustration of how the metabolic activity of specific genera or species in the gut microbiota was analyzed by flow cytometry. **B** Flow cytometry dot plots showing the relative abundances and FADA labeling intensities of two bacterial genera in the microbiota after staining with corresponding FISH probes

21 The strategy was named "MeDabLISH" (Metabolic D-amino acid-based Labeling and In Situ Hybridization-facilitated Probing), whose schematic illustration was shown in Fig. 3A. The metabolic activity analysis data of two genera in the gut microbiota were depicted in Fig. 3B.

## DISCUSSION

The paper introduces a streamlined strategy that leverages the synergy of FDAA *in vivo* metabolic labeling and FISH to investigate the *in situ* growth of gut bacteria within the complex intestinal environment. Further examples of bacterial imaging results can be found in our previously published studies (Lin *et al.* 2020a, 2020b, 2021b). By employing STAMP labeling, this method effectively reveals the growth and division dynamics of both cultivable and previously uncultivable gut bacteria, while FISH helps in species identification. The proposed strategy eliminates the need for complex isolation and *in vitro* bacterial culture procedures, enabling direct examination of gut bacteria's physiological behaviors *in situ*. This approach offers new insights and methodologies for exploring the largely unexplored gut bacteria's basic microbiology. Additionally, the technique enables the simultaneous detection of numerous species within the ecosystem, markedly enhancing the efficiency of bacterial morphology studies.

Furthermore, the integration of FDAA and FISH introduces a new quantitative technique for assessing the metabolic activity of diverse gut bacteria. Compared to the peak-to-trough ratio (PTR) method (Joseph *et al.* 2022; Korem *et al.* 2015), which indirectly estimates microbial growth rates by calculating the ratio of DNA copy numbers near the replication initiation and termination sites, the MeDabLISH approach provides a more intuitive and straightforward reflection of the *in vivo* metabolic activities and growth dynamics of gut bacteria. This method is also expected to be applicable in other areas of microbiota research, such as quantitatively examining the metabolic activity changes of gut bacteria in response to treatments with drugs, prebiotics, and immunomodulatory factors that can influence microbial community activities. In conclusion, this protocol employs the newly developed methodology to shed light on the “dark matter” of the gut, overcoming the existing limitations in directly observing and measuring the *in vivo* growth of gut bacteria.

## Conflict of interest

Yingjun Zhou, Liyuan Lin and Wei Wang declare that they have no conflict of interest.
